# The Farming of Patients

**Published:** 1892-02-13

**Authors:** 


### The Doctor's Armchair.

### THE FARMING OF PATIENTS.

A circular was received through the post by the writer a few days ago which caused him some astonishment. The presumption is that it had been sent to the members of the medical profession as a body, or to selected individuals, since it is a printed document, setting forth in the usual business terms certain advantages which medical men may secure under certain conditions. The relations of doctors with their patients are peculiar, implying much loyalty on the one side, and much confidence on the other. It is absolutely necessary in the interests of sick persons that those relations should continue and be constantly improved. The medical man who takes upon himself the care of a sick fellow creature should have one purpose in life with regard to that fellow creature, and one only, viz., the speedy and economical restoration of the sick person to perfect health. He should have what has been well called "a single eye" to that sole object. If any other motive, such for example as the prolonging of attendance to secure the greater profit, or the supplying unnecessary medicines or foods in order to advantage himself or a third party, be in the doctor's mind, he ceases at once to act as the loyal friend of his patient, and becomes recreant and odious.

These are, in a brief form, the general principles which should guide a medical man in his conduct towards his patients; and a twenty years' experience of medicine justifies the assertion that they are the actual principles which guide the majority of British medical men all over the country. It is proper to bring to the touchstone of these principles the circular to which the writer has already alluded, and which he supposes that at least a large number of medical men have received in common with himself.

The circular sets forth that a private asylum and retreat for middle-class patients is to be formed, or has already been formed, in a certain division of Great Britain. The promoters desire the co-operation of medical men; and in order that they may secure that co-operation, offer to the members of the profession founders' shares, *without any payment whatever*. This is the first point to which the attention of readers is called. The next point is the peculiar conditions under which the free gift founders' shares are to be held. Those shares involve no liability whatever, beyond the possible payment of a single shilling; and yet at the yearly distribution of profit a dividend would be paid, based upon the amount of profit which had accrued from patients introduced by holders of founders' shares. Those holders of founders' shares not having sent any patients, or developed the work of the institution, will receive no benefit thereupon, and the whole benefit will be received by those persons who have introduced patients thereto. "This idea," says the circular, "we believe, is novel, and yet at the same time its feasibility must necessarily present itself to you on carefully considering the method of appropriation of the revenue."

That is a fair statement of the case from the point of view of the promoters of this "novel" institution. Now let us consider the question from the standpoint of medical honesty and loyalty to patients. The circular, first of all, offers a bait to members of the medical profession which is constructed with a great show of skill. "Founders' shares," and founders' shares for nothing; it looks tempting enough even for a medical salmon to swallow. That is the bait: but where will the hook land us if the bait be swallowed? It will land us in the midst of three separate temptations, each more disgraceful than its predecessor. The first temptation is, that since we are to have founders' shares and profits in a lunatic asylum we shall be anxious to discover signs of lunacy in as many of our patients as possible. The second is, that having profit-bearing shares in one particular asylum and in no other, we shall be under a strong inducement to send patients to that asylum and nowhere else, whether it be the best or the worst managed institution in the world. The third is, that if we once send patients to any such institution we shall have the dangerous motive of keen self-interest constantly prompting us to keep them there as long as possible.

Of course a bait like this, which shows the hook so plainly, can be no temptation to doctors of honourable character, or even to those who have more than the average school-boy's knowledge of the world to guide them. But there are men in the ranks of medicine who may not see the obvious drift of the whole business; who may, indeed, be foolish enough to imagine that it would be a right and proper thing for them to have shares in a private lunatic asylum, and so to preserve a kind of indirect control over any of their patients who may be so unfortunate as to become insane. Such an idea cannot be too strongly denounced. It ought to be every doctor's aim, as it is every doctor's interest, to keep his patients outside lunatic asylums. The time may come when such institutions will be looked upon as hospitals, and when no stigma of any kind will attach to persons who have once been placed for cure within their walls. But as things are at present, it is eminently desirable that all mental patients who can, with safety to themselves and others, be treated by their ordinary practitioners at their own homes, or at the sea-side or elsewhere, under proper control, should be so treated. Many lunacy cases recover much more quickly outside an asylum than inside, and their restored sanity is much more lasting. Patients who have been saved from what they consider the stigma of an asylum often feel towards their medical attendants a degree of gratitude, affection, and confidence which are both touching in themselves and of the greatest possible value as elements of cure in possible future attacks.

Any kind of "farming" of patients, or, indeed, the mere appearance of such farming, is utterly repugnant to the medical man who is possessed of the right instincts of his profession. But when the farming is proposed to be done at the expense of lunatics whom the State permits us to imprison, not for a week, but for a life time, then even the mere temptation to such farming becomes revolting and horrible. There is a sense, no doubt, in which the profession of medicine is our "business," and the business by which we live. But a stern retribution awaits the man who has for his controlling motive in his medical work the making of as much money as possible. The one thing for a doctor to do is to cure his patient, or, at least, to help him, and that as promptly, as kindly, and even as economically as he can. His reward, he must get as he may. If he does not get it in this world, he must hope for it in the next; and if he has no hope of it in the next, he must try to do without it. There are worse ways of spending one's life than that of serving one's fellow-men first and making the question of reward a secondary consideration.

